# Floral traits of mammal‐pollinated *Mucuna macrocarpa* (Fabaceae): Implications for generalist‐like pollination systems

**DOI:** 10.1002/ece3.4404

**Published:** 2018-07-30

**Authors:** Shun Kobayashi, Tetsuo Denda, Chi‐Cheng Liao, Yu‐Hsiu Lin, Shu‐Hui Wu, Masako Izawa

**Affiliations:** ^1^ Faculty of Science University of the Ryukyus Nishihara Okinawa Japan; ^2^ Department of Life Science Chinese Culture University Taipei Taiwan; ^3^ Endemic Species Research Institute Nantou Jiji Taiwan; ^4^ Taipei Botanical Garden Taiwan Forestry Research Institute Taipei Taiwan

**Keywords:** floral shape, mammalian pollinator, *Mucuna macrocarpa*, nectar, pollination, regional differences

## Abstract

Floral traits are adapted by plants to attract pollinators. Some of those plants that have different pollinators in different regions adapt to each pollinator in each region to maximize their pollination success. *Mucuna macrocarpa* (Fabaceae) limits the pollinators using its floral structure and is pollinated by different mammals in different regions. Here, we examine the relationships between floral traits of *M. macrocarpa* and the external morphology of mammalian pollinators in different regions of its distribution. Field surveys were conducted on Kyushu and Okinawajima Island in Japan, and in Taiwan, where the main pollinators are the Japanese macaque *Macaca fuscata*, Ryukyu flying fox *Pteropus dasymallus*, and red‐bellied squirrel *Callosciurus erythraeus*, respectively. We measured the floral shapes, nectar secretion patterns, sugar components, and external morphology of the pollinators. Results showed that floral shape was slightly different among regions and that flower sizes were not correlated with the external morphology of the pollinators. Volume and sugar rate of nectar were not significantly different among the three regions and did not change throughout the day in any of the regions. However, nectar concentration was higher in Kyushu than in the other two regions. These results suggest that the floral traits of *M. macrocarpa* are not adapted to each pollinator in each region. Although this plant limits the number of pollinators using its flower structure, it has not adapted to specific mammals and may attract several species of mammals. Such generalist‐like pollination system might have evolved in the Old World.

## 1 INTRODUCTION

Plants those strongly depend on specific pollinators for pollination success adapt their floral traits to the characteristics of their pollinators. For example, previous studies have demonstrated that the flowering season matches the season when pollinators appear (Fleming, Sahley, Nolland, Nason, & Hamrick, 2001; Peter & Johnson, 2014; Queiroz, Quirino, Lopes, & Machado, 2016), that floral shapes fit the external characteristics of pollinators (Boberg et al., 2014; Nagano et al., 2014; Nillson, 1988), that floral colors match the color vision of pollinators (Hoballah et al., 2007; Levin & Kerster, 1967; Sobel & Streisfeld, 2015), and that attractive traits of nectar secretion and odor emission patterns fit the activity patterns of pollinators (Cruz‐Neto, Machado, Galetto, & Lopes, 2015; Heath, Landolt, Dueben, & Lenczewski, 1992; Krömer, Kessler, Lohaus, & Schmidt‐Lebuhn, 2001). When the floral shape fits the external morphology of its pollinator, more pollen grains adhere to fixed positions on the pollinator, so that the plant transfers more pollen effectively (Bloch & Erhardt, 2008; Campbell, Waser, & Price, 1996). When the nectar secretion and odor emission patterns fit the activity patterns of pollinators, the plant can attract its pollinators more effectively, and the pollinator may visit more frequently, presumably increasing pollination success (Gijbels, van den Ende, & Honnay, 2014; Leiss & Klinkhamer, 2005).

Previous studies showed that pollinators induce selective pressures on floral traits. Plant adaptations to specific pollinators can occur when their distribution ranges closely overlap with those of their pollinators. However, when the distributional ranges of plants do not overlap with those of their specific pollinators, some plants change their flowering phenology, shape, color, nectar secretion pattern, and/or volatile components to attract another pollinator (Boberg et al., 2014; Nagano et al., 2014; Sun, Gross, & Schiestl, 2014). Finally, plants may speciate in each site (Fleming et al., 2001; Forest et al., 2014; Gowda & Kress, 2013). Otherwise, a plant might adapt its floral traits to several pollinator species (i.e., become generalized).

Insect‐ or bird‐pollinated plants have been targeted in previous studies, and no studies have investigated the adaptations of mammal‐pollinated plants to different mammalian pollinators in different regions. Although the diversity of mammal‐pollinated plants is relatively low compared with insect‐pollinated plants, there are many mammal‐pollinated species throughout the world, especially in the tropics (Carthew & Goldingay, 1997; Fleming & Kress, 2013). However, pollination ecology of mammal‐pollinated plants has been studied mainly in Australia and Africa, with only a few studies in Asia (Willmer, 2011).

*Mucuna macrocarpa* (Fabaceae) is a woody vine plant which is distributed from Southeast Asia to Kyushu, Japan (Tateishi & Ohashi, 1981). This flower is papilionaceous and utilizes a special pollination stage called “explosive opening” (Toyama, Kobayashi, Denda, Nakamoto, & Izawa, 2012). Stamens and pistils are tightly enclosed by the keel petals and are exposed when the flower opens explosively. The explosive opening is facilitated by different mammals (explosive openers) in different regions: Japanese macaques (*Macaca fuscata*) and Japanese martens (*Martes melampus*) in Kyushu; Ryukyu flying foxes (*Pteropus dasymallus*) on Okinawajima Island; and red‐bellied squirrels (*Callosciurus erythraeus*), Formosan striped squirrels (*Tamiops maritimus*), and masked palm civets (*Paguma larvata*) in Taiwan (Kobayashi, Denda, et al., 2015; Kobayashi et al. 2017; Toyama et al., 2012). Among them, Japanese macaques and red‐bellied squirrels open the most flowers in Kyushu and Taiwan, respectively, compared with other explosive openers. Thus, these two species are the main pollinators in these regions (Kobayashi, Denda, et al., 2015; Kobayashi et al. 2017). Except for Japanese macaques, all explosive openers hold the wing petals with their forelegs and insert their snouts into the gap between the wings and banner petals and then push up the banner using their snout to feed on the nectar located inside the calyx. Japanese macaques open flowers using their hands. When all explosive openers open flowers successfully, many pollen grains adhere to them. In addition, the explosive opening is an indispensable stage for fruit set, as flowers do not open by themselves and no fruits are observed on unopened flowers (Kobayashi, 2017). Although bees collect pollen and stigma attaches to their body in some cases, most pollen grains are removed by explosive openers (Kobayashi, Denda, et al., 2015; Kobayashi et al. 2017; Toyama et al., 2012). Thus, explosive openers are considered as the main pollinators (Kobayashi, Denda, et al., 2015; Kobayashi et al. 2017; Toyama et al., 2012).

In this study, we aimed to reveal the relationships between floral traits (shape and nectar) and characteristics of explosive openers (external morphology and daily activity patterns) in three regions. We examined the following hypotheses: (1) Floral shapes differ among the three regions; (2) face size, which is an important trait for opening flowers explosively, is different among explosive openers; (3) floral shape correlates with face size of explosive openers; (4) nectar secretion patterns differ among the three regions; and (5) nectar secretion patterns correlate with the activity patterns of the main explosive openers.

## 2 MATERIALS AND METHODS

### 2.1 Study sites

**Figure 1:**
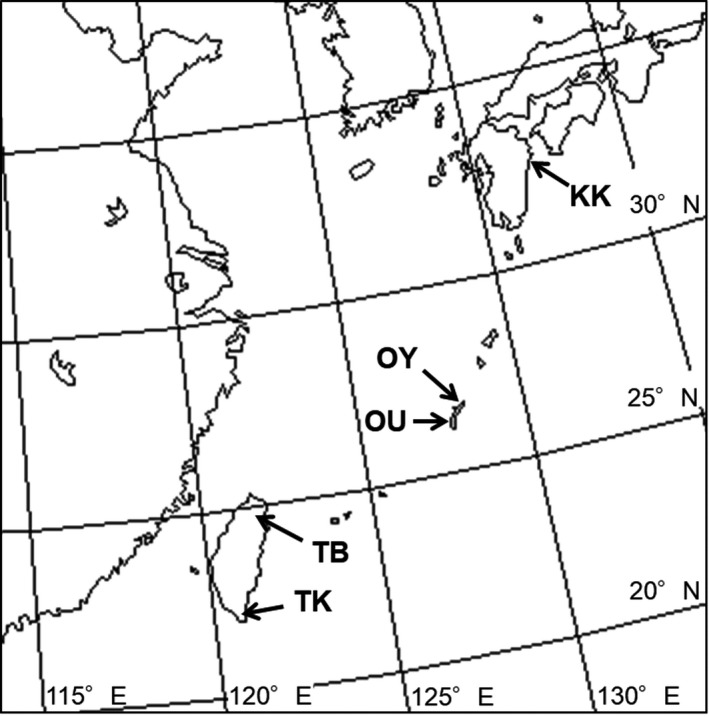
Map of study sites. KK: Kyushu, OY: northern site on Okinawajima Island, OU: southern site on Okinawajima Island, TB: northern site in Taiwan, TK: southern site in Taiwan

This study was conducted from 2013 to 2016 at five sites in three different regions with different explosive openers: one site in Kyushu (KK: 32°45–46′ N, 131°51–52′ E), two sites on Okinawajima Island (northern site [OY]: 26°46–49′ N, 128°14–17′ E and southern site [OU]: 26°14′ N, 127°45′ E) in Japan, and two sites in Taiwan (northern site [TB]: 24°38–41′ N, 121°22–25′ E and southern site [TK]: 21°57′ N, 120°48′ E) (Figure 1). Kyushu is a large island, but distribution of *M. macrocarpa* is limited to the study site. This species grows in the southward steep slope (Kobayashi, Izawa, et al., 2015). The annual mean temperature (2008–2017) in Kamae, the nearest meteorological observation point, is 17.4°C, and annual mean precipitation is 2,401 mm (Japan Meteorological Agency, 2018). On Okinawajima Island, this species is distributed throughout the island and mainly grows along the valley (Kusumoto & Enoki, 2008). The annual mean temperature (2008–2017) in Oku, the nearest meteorological observation point of OY, is 20.8°C, annual mean precipitation is 2,611 mm, and those in Naha, the nearest meteorological observation point of OU, are 23.4°C and 2,159 mm (Japan Meteorological Agency, 2018). In Taiwan, this species is also distributed from north to south, but elevation of distribution area is ranging from 100 to 1,500 m (Herbarium of National Taiwan University, 2012). Annual mean temperature (2010–2017) in Fu Xing, the nearest meteorological observation point of TB, is 20.0°C and mean annual precipitation is 3,194 mm, while those in Heng Chun, the nearest meteorological observation point of TK, are 25.8°C and 2,166 mm (Central Weather Bureau, 2018). All study sites are evergreen forests, but the flora is quite different among these sites. In addition, the individual planted in TK originated from near the study site.

### 2.2 Measurements of flowers and explosive openers

**Figure 2:**
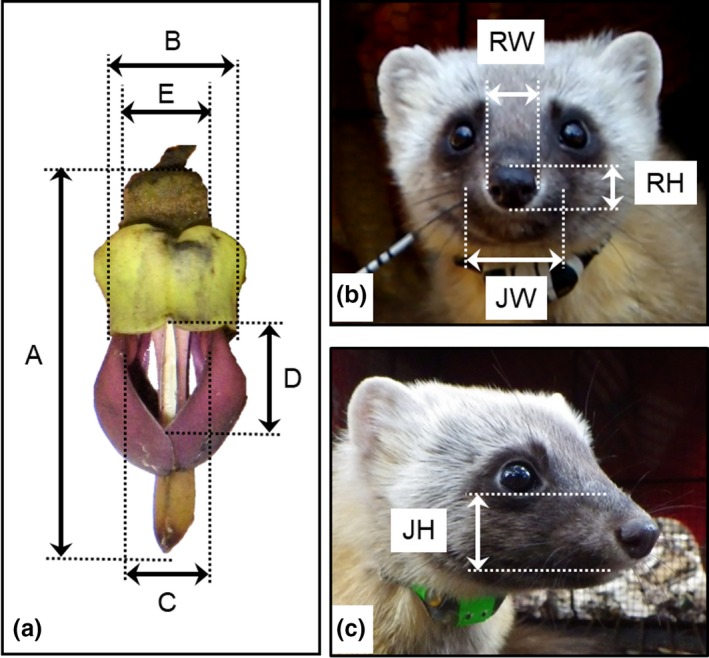
Measured parts of flowers (a) and explosive openers (b, c). A: flower length, B: gap between banner petals, C: gap between wing petals, D: gap between the top of the banner and top of the wings, E: width of calyx, RW: width of rhinarium, RH: height of rhinarium, JW: width of upper jaw at the tip of the lower jaw, and JH: height of upper jaw at the tip of the lower jaw

Firstly, floral shape and size were compared among regions. Mature and dropped fresh flowers were collected from four individuals in Kyushu, six individuals (OY: 3, OU: 3) on Okinawajima, and eight individuals (TB: 7, TK: 1) in Taiwan. Flower length, the gap between banner petals, gap between wing petals, gap between the top of the banner and the top of wings, and the width of the calyx were measured (Figure 2a). Floral shape was compared among regions using principal component analysis (PCA).

Secondly, the height and width of the rhinarium and height and width of parts of the upper jaw at the top of the lower jaw of explosive openers were measured for external morphology of mammalian pollinators in each region (Figure 2b,c). Measured species were Japanese martens, Ryukyu flying foxes, red‐bellied squirrels, Formosan striped squirrels, and masked palm civets, which use their snouts for explosive opening (Kobayashi, Denda, et al., 2015; Kobayashi et al. 2017; Toyama et al., 2012). Japanese macaques were the main openers in Kyushu; however, as they opened flowers using their hands, we did not measure any facial parts. Samples of Japanese martens were those killed by cars, which were stored in the freezer (*n *=* *16), and live individuals captured for ecological research (*n *=* *10) on the Tsushima Islands, Japan. Captive Ryukyu flying foxes at the University of the Ryukyus were measured (*n *=* *16). Eleven were rescued from the wild, and five were born in captivity. Specimens of red‐bellied squirrels (*n *=* *31) and masked palm civets (*n *=* *17) stored in the National Museum of Natural Science of Taiwan and Endemic Species Research Institute were measured. We could not find available specimens of striped squirrels, so we used the mean value of skull data (*n *=* *4) measured by Chang (2011). The external morphology of opener snouts was compared using PCA.

Finally, the relationship between the floral gap size and snout size of the explosive openers was investigated in each region. When the explosive openers opened flowers using their snouts, the direction of snout insertion into the flower gap was almost fixed in the right‐side upward direction (see Kobayashi, Denda, et al., 2015; Kobayashi et al. 2017; Toyama et al., 2012). Thus, the gaps of flower banners were compared using the width of rhinarium or width of upper jaw of openers, and the gaps between the tops of banners and the tops of flower wings were compared using the height of rhinarium or height of upper jaw of openers (see Figure 2).

### 2.3 Measurement of nectar

To investigate the daily change in nectar secretion, the volume, weight, and sugar concentration of nectar were measured every 3 hrs. Mature flowers were picked from four individuals in Kyushu, five individuals (OY: 1, OU: 4) on Okinawajima and six individuals (TB: 5, TK: 1) in Taiwan. Nectar was collected from inside the calyx, and volume was measured using a microsyringe (MS‐N100; Ito Corporation, Tokyo, Japan). Nectar weight was measured using a digital portable balance (TR‐SC30; Pepaless, Hyogo, Japan) and sugar concentration (Brix index) was measured using a handheld refractometer with special compensating thermometer (HSR‐500; Atago, Tokyo, Japan).

In addition, the sugar composition was analyzed using high‐performance liquid chromatography (HPLC). Nectar used for HPLC was collected from 12 flowers at 0900 and 2100 hr in each region and put into microtubes and stored in the freezer (−20°C) until analysis. Firstly, the acetonitrile solution (nectar:distilled water:acetonitrile = 2:33:65) was prepared. Then, the acetonitrile solution was percolated using a Mini‐UniPrep syringeless filter (UN203NPUAQU; GE Healthcare companies, Buckinghamshire, UK). The percolated acetonitrile solution was analyzed using an HPLC analysis machine (LC‐20AD; Shimadzu Corporation, Kyoto, Japan). The Sugar‐D column (Nacalai Tesque, Kyoto, Japan) was used, and 80% acetonitrile solution was delivered at a flow rate of 0.5 ml/min. Sugar was identified from a chromatogram, which had been calculated previously from the components of each sugar found in our samples.

ANOVA was used to compare the volume, weight, and sugar concentration of nectar in flowers from each region, and the chi‐square test was used to compare the sugar components over time and across regions. All statistical analyses were conducted using the statistical software R ver. 3.4.0 (R Core Team, 2017).

## 3 RESULTS

### 3.1 Comparison between floral and opener shapes

**Figure 3:**
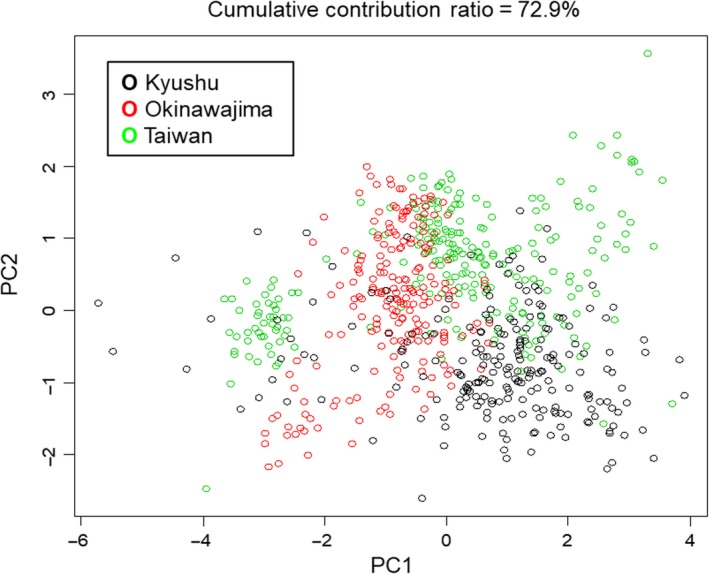
Principal component analysis of floral shape. PC1 indicates the size of flowers, and PC2 indicates the gap size of banners

**Figure 4:**
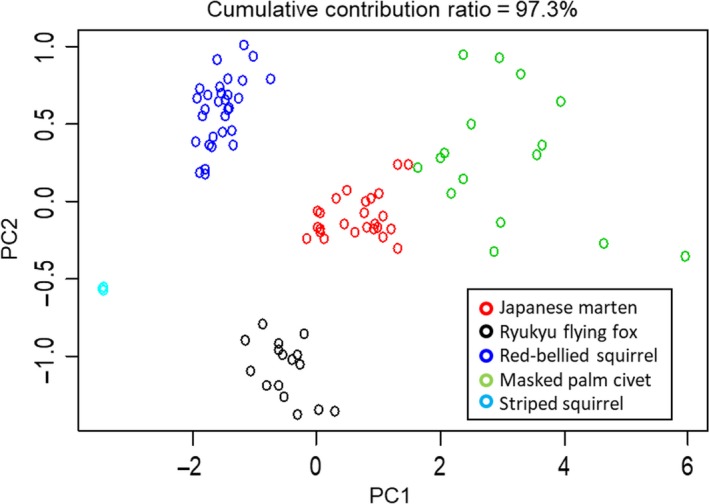
Principal component analysis of shape of the tip of the nose of explosive openers. PC1 indicates the size of the tip of the nose, and PC2 indicates the height‐to‐width ratio

All flower parts were significantly different among regions, with the longest length observed in Kyushu and the shortest on Okinawajima (ANOVA for each flower trait; *p *<* *0.05) (Table 1). The maximum and minimum values of each part were observed in the different regions. The PCA of floral parts showed that floral shape had the least difference among the three regions (Figure 3). In addition, the external morphology of the openers’ snouts differed among the three regions. Snouts of Japanese martens and masked palm civets were larger than those of squirrels (Figure 4). Snouts of Ryukyu flying foxes were medium‐sized; however, the rhinarium was larger than that of any other opener (Figure 4).

**Table 1:** Mean (±*SD*) floral characteristics (mm) in each region. Measured parts are shown in Figure 1

	Kyushu (*n *=* *243)	Okinawajima (*n *=* *206)	Taiwan (*n *=* *221)	ANOVA	
				*F* value	*p* value
Length of flower	76.66 ± 2.32	69.67 ± 1.83	73.62 ± 6.83	132.93	<0.05
Width of banner	24.82 ± 2.58	26.28 ± 2.78	26.84 ± 2.77	33.61	<0.05
Gap between wings	16.15 ± 3.34	14.20 ± 2.22	13.21 ± 2.45	69.61	<0.05
Width between top of banner and top of wings	30.09 ± 5.00	26.09 ± 3.75	31.43 ± 5.63	69.78	<0.05
Width of calyx	20.09 ± 2.22	18.28 ± 1.00	18.83 ± 1.22	74.88	<0.05

**Figure 5:**
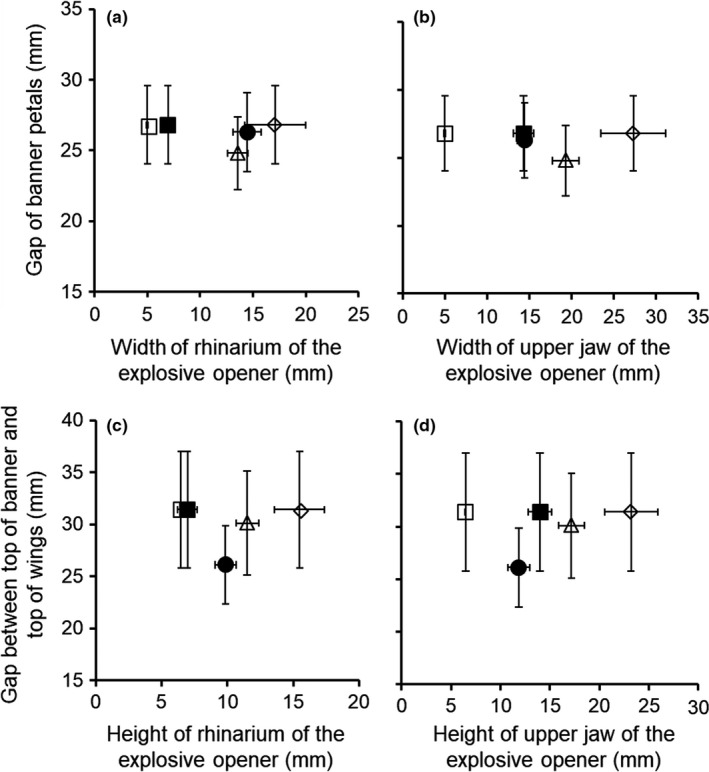
Comparisons between snout sizes of explosive openers and gaps of flowers in each region. (a) Width of rhinarium versus gap between banner petals; (b) width of upper jaw versus gap of banner petals; (c) height of rhinarium versus gap between the top of the banner and top of the wings; (d) height of upper jaw versus gap between the top of the banner and top of the wings. △: Japanese marten, ●: Ryukyu flying fox, ■: red‐bellied squirrel, □: striped squirrel, ◇: masked palm civet. Filled marks indicate the main openers (pollinators), and open marks indicate nonmain openers. Error bars indicate *SD*

The snout sizes of Ryukyu flying foxes and red‐bellied squirrels were smaller than the flower gaps (gaps between banner petals and gaps between the tops of the banner and the tops of wings; see Figure 2a) into which explosive openers inserted their snouts (Figure 5). The rhinarium sizes of Japanese martens and masked palm civets were smaller than their respective flower gaps; however, their upper jaw sizes were larger than the flower gaps (Figure 5). The mean size of the flower gap of flower and the mean size of the snout of the main explosive openers were not positively correlated with each other (Figure 5a–c), except for height of upper jaw versus gap between the top of the banner and top of the wings (Figure 5d), although flower gaps showed high variation in all regions (Figure 5).

### 3.2 Nectar production pattern

**Figure 6:**
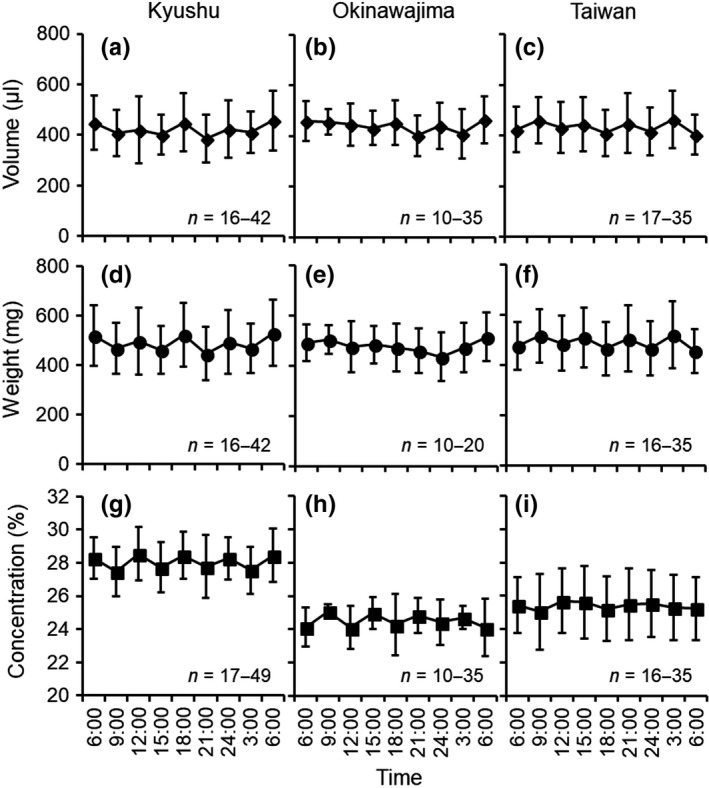
Nectar volume, weight, and concentration in flowers from Kyushu (a, d, and g), Okinawajima (b, e, and h), and Taiwan (c, f, and i). Error bars indicate *SD*

The nectar volume, weight, and nectar concentration did not change throughout the day in any of the regions (ANOVA; *p *>* *0.05) (Table 2, Figure 6). Also, nectar was stored inside the calyx throughout the day. Nectar volume was 429.4 ± 112.14 (mean ± *SD*), 437.1 ± 86.6, and 429.5 ± 99.2 μl, and weight was 499.1 ± 126.4, 489.9 ± 91.2, and 484.8 ± 112.0 mg in Kyushu, Okinawajima, and Taiwan, respectively; neither were different among the three regions (ANOVA; volume: *F*_2,715_ = 0.41, *p *=* *0.67, weight: *F*_2,635_ = 1.02, *p *=* *0.36) (Figure 6). However, nectar concentration was 28.2 ± 1.5% in Kyushu, which was significantly higher than that in Okinawajima (24.5 ± 1.4%) and Taiwan (25.4 ± 2.0%) (ANOVA; *F*_2,721_ = 330.97, *p *<* *0.05) (Figure 6).

**Table 2:** Results of ANOVA comparing among sampling times of nectar

	Kyushu			Okinawajima			Taiwan		
	*n*	*F* value	*p* value	*n*	*F* value	*p* value	*n*	*F* value	*p* value
Volume	275	1.33	0.23	198	0.73	0.66	245	1.19	0.31
Weight	274	1.53	0.15	120	0.55	0.82	244	1.2	0.29
Concentration	283	1.93	0.06	197	0.91	0.51	244	0.28	0.97

**Figure 7:**
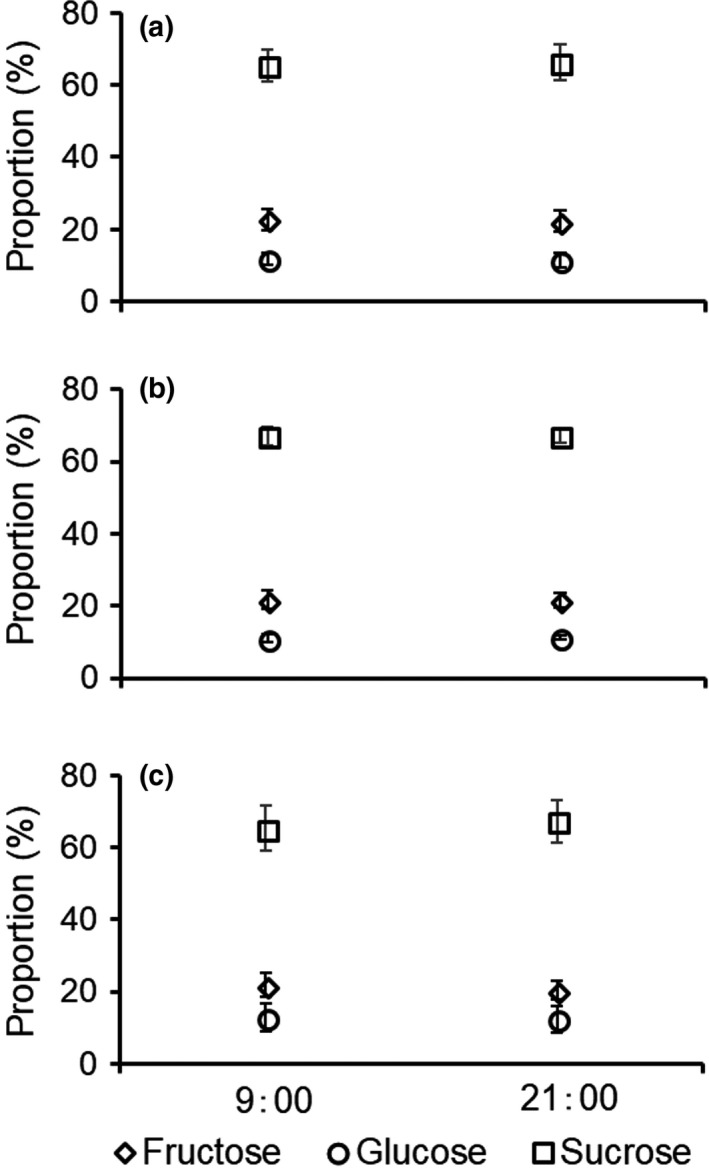
Proportions of sugar components in nectar of flowers from Kyushu (a), Okinawajima (b), and Taiwan (c) at 0900 and 2100 hr. Error bars indicate standard deviation

The analysis of sugar component ratio showed that sucrose was dominant in all regions. The ratio of sucrose was over 65%, and the range of mean sugar ratio was 1.93–2.07 in the three regions (Figure 7). In addition, the sugar component ratio between morning and night was the same in all regions (chi‐square test; Kyushu: *χ* ^2^_2_ = 0.00, *p* > 0.05, Okinawajima: *χ* ^2^_2_ = 0.00, *p *>* *0.05, Taiwan: *χ* ^2^_2_ = 0.05, *p *>* *0.05) (Figure 7).

## 4 DISCUSSION

Floral shape and size of *M. macrocarpa* differed among the three regions. Although the sample size of striped squirrel was small, size and external morphology of snouts of explosive openers were different; however, the sizes of flower gaps and the rhinarium size of the main explosive openers were not correlated. Nectar was stored inside the calyx throughout the day, and daily nectar secretion patterns were not different among regions. Therefore, these results confirmed hypotheses 1 and 2, while hypotheses 3, 4 and 5 were rejected.

Many previous studies clarified that some plants adapt their floral shapes to the main pollinators in each region when the main pollinator differs among regions (Anderson & Johnson, 2008; Boberg et al., 2014; Johnson & Steiner, 1997). In this study, the floral shape and size of *M. macrocarpa* slightly differed among regions. In addition, there were specific mammalian openers in each region (Kobayashi, Denda, et al., 2015; Kobayashi et al. 2017; Toyama et al., 2012), and snout sizes of these openers were also different among regions. However, contrary to our third hypothesis, floral dimensions did not correlate with snout sizes of main openers. Thus, we concluded that this plant did not adapt its floral shapes to individual pollinators. One possible reason might be that mammals have a higher intraspecific size variation (including differences between sexes or among ages) than insects or birds and cannot exert sufficient selection pressure on the plants they pollinate.

Nectar secretion patterns were not adapted to mammalian openers in each region in this study. Bat‐pollinated plants generally secrete nectar at night (Fægri & van der Pijl, 1979; Willmer, 2011). This characteristic is also observed in *Mucuna* species (Table 2); for example, *M. urens*, whose explosive opening depends on tiny nectar‐feeding bats (*Glossophaga soricina*), secretes nectar only at night (Agostini, Sazima, & Galetto, 2011). In contrast, *M. japira* and *M. sempervirens*, which are explosively opened by passerine birds (*Cacicus haemorrhous*) and squirrels (*Dremomys pernyi* and *C. erythraeus*), respectively, secrete nectar throughout the day (Agostini et al., 2011; Liu, Shah, Zha, Mohsin, & Ishtiaq, 2013) (Table 3). However, contrary to our fourth and fifth hypotheses, the nectar secretion patterns of *M. macrocarpa* did not coincide with the activity patterns of each explosive opener in each region. Furthermore, nectar secretion patterns were not different among regions even when either diurnal or nocturnal pollinators occurred (Kobayashi, Denda, et al., 2015; Kobayashi et al. 2017; Toyama et al., 2012). In comparison, plants pollinated by pteropodid bats were reported to have higher sucrose content (Baker & Baker, 1983; Baker, Baker, & Hodges, 1998), which might also be the case in the nonflying mammal‐pollinated plants, although the sample size of previous studies is small (Willmer, 2011). The concentration and sugar ratio of nectar in *Mucuna* are determined by the species of pollinator (Agostini et al., 2011; Liu et al., 2013), except for *M. macrocarpa* (Table 3). Although the squirrel‐pollinated *M. sempervirens* has some similarities in nectar secretion patterns (i.e., nectar concentration, sugar ratio, and daily variation) (Table 3), the nectar secretion patterns of *M. macrocarpa* are unique among *Mucuna* species. In conclusion, *M. macrocarpa* may attract both diurnal and nocturnal mammals. Although *M. macrocarpa* uses explosive openers, a special pollination mechanism, and restricts pollinator species, the floral shapes and nectar secretion dynamics do not match the main explosive openers in each region. Therefore, mammalian pollinators may not be selective pressures for floral traits in this plant.

**Table 3:** Comparison of components of nectar secretion patterns among species

Species	Study sites	Explosive opener	Volume (μl)^a^	Weight (mg)^a^	Concentration (%)^a^	Dominant sugar	Sugar ratio S/(F + G)^a^	Secretion pattern
*M. macrocarpa* b	Kyushu	Japanese macaque (Japanese marten)	429.4	499.1	28.2	Sucrose	1.93	Throughout the day
	Okinawajima	Ryukyu flying fox	437.1	489.9	24.5	Sucrose	2.07	Throughout the day
	Taiwan	Red‐berried squirrel (striped squirrel/masked palm civet)	429.5	484.8	25.4	Sucrose	1.97	Throughout the day
*M. sempervirens* c	China	Squirrels	20–80	—	29	Sucrose	5.38	Throughout the day
*M. urens* d	Brazil	Tiny bats	310	—	16	Constant	0.37	Night only
*M. japira* d	Brazil	Passerine bird	340	—	10	Fructose	0.26	Throughout the day

^a^Each value shows average. ^b^Present study. ^c^Liu et al. (2013). ^d^Agostini et al. (2011).

Although the floral traits of *M. macrocarpa*, such as shape and nectar secretion patterns, did not match the main pollinators in each region, there were small differences among regions. Floral traits only differed in biotic factors, but also in abiotic factors (e.g., Campbell, 1996; Petanidou, Goethals, & Smets, 2000). Therefore, abiotic factors, such as temperature, precipitation, and soil conditions, should be considered as well as genetic drift caused by bottlenecks and/or founder effects on floral traits in future.

In the eastern Caribbean, bird‐pollinated plants have different pollinators in different regions and have evolved their floral traits depending on the traits of the pollinator in each region (Gowda & Kress, 2013; Temeles & Kress, 2003). In the Sonoran Desert, bat‐pollinated cactus species are known to adapt to both bird and bat pollinators (i.e., become more generalized) (Fleming et al., 2001). Examples of vertebrate pollinator shifts are reported from the New World, and these plants adapt to their new bird pollinators through speciation. However, no reports have demonstrated that mammal‐pollinated plants have different mammalian pollinators in different regions, except for *M. macrocarpa*. The *Mucuna* group is distributed throughout tropics and subtropics (Schrire, 2005), and many species are pollinated specifically by birds or bats (Agostini, Sazima, & Sazima, 2006; Cotton, 2001; Grünmeier, 1993; Hopkins & Hopkins, 1993; Sazima, Buzato, & Sazima, 1999). However, plants pollinated by multiple mammals are found only in Asia (Kobayashi, Denda, et al., 2015; Kobayashi et al. 2017; Toyama et al., 2012). One reason why bat‐pollinated plants have become specialized or adapted to local vertebrate pollinators in the New World is the high diversity of nectar‐feeding specialist birds and bats (Fleming & Kress, 2013). Conversely, the diversity of nectar‐feeding mammals is low in the Old World, especially in Asia (see Fleming & Kress, 2013), while the diversity of omnivorous mammals, such as squirrels, macaques, and civets, is high in Southeast Asia (Corlett, 2007). Therefore, in case the nectar‐feeding specialist bats and birds are lacking, plants have evolved nonflying mammal‐dependent and/or generalist‐like pollination systems.

However, this hypothesis is based on the information of one specific genus. Mammalian pollinators are not well clarified in Asian regions. Further researches clarifying pollinators of mammal‐pollinated plants are needed to establish this hypothesis.

## CONFLICT OF INTEREST

None declared.

## AUTHOR CONTRIBUTIONS

S. K., T. D., and M. I. conceived and designed the study. S. K. contributed to all surveys and analysis. T. D. and M. I. surveyed in Okinawajima, and C.‐C. L. and S.‐H. W surveyed in Taiwan. All authors contributed to make up the draft and approved for publication.

## DATA ACCESSIBILITY

Data available from the Dryad Digital Repository: https://doi.org/10.5061/dryad.nv5cr00
